# BAGET 2.0: an updated web tool for the effortless retrieval of prokaryotic gene context and sequence

**DOI:** 10.1093/bioinformatics/btab082

**Published:** 2021-02-03

**Authors:** Benjamin Hepp, Violette Da Cunha, Florence Lorieux, Jacques Oberto

**Affiliations:** Université Paris-Saclay, CEA, CNRS, Institute for Integrative Biology of the Cell (I2BC), 91198, Gif-sur-Yvette, France; Université Paris-Saclay, CEA, CNRS, Institute for Integrative Biology of the Cell (I2BC), 91198, Gif-sur-Yvette, France; Université Paris-Saclay, CEA, CNRS, Institute for Integrative Biology of the Cell (I2BC), 91198, Gif-sur-Yvette, France; Université Paris-Saclay, CEA, CNRS, Institute for Integrative Biology of the Cell (I2BC), 91198, Gif-sur-Yvette, France

## Abstract

**Motivation:**

The retrieval of a single gene sequence and context from completely sequenced bacterial and archaeal genomes constitutes an intimidating task for the wet bench biologist. Existing web-based genome browsers are either too complex for routine use or only provide a subset of the available prokaryotic genomes.

**Results:**

We have developed BAGET 2.0 (Bacterial and Archaeal Gene Exploration Tool), an updated web service granting access in just three mouse clicks to the sequence and synteny of any gene from completely sequenced bacteria and archaea. User-provided annotated genomes can be processed as well. BAGET 2.0 relies on a local database updated on a daily basis.

**Availability and implementation:**

BAGET 2.0 befits all current browsers such as Chrome, Firefox, Edge, Opera and Safari. Internet Explorer 11 is supported. BAGET 2.0 is freely accessible at https://archaea.i2bc.paris-saclay.fr/baget/

## 1 Introduction

The completely sequenced prokaryotic genomes from the National Center for Biotechnology Information (NCBI) constitute an indispensable and freely available source of information for a number of biological disciplines. Several desktop programs provide local access to nucleotide-level information from complete genomes but require installation in addition to the download, formatting and storage of large genomic files. These include the free BAC-BROWSER (Garanina *et al.*, 2018) and the commercially available SnapGene (www.snapgene.com) or Geneious (Kearse *et al.*, 2012). To overcome these limitations, several web-based genome browsers such as Gbrowse (Stein, 2013), Ensembl (Yates *et al.*, 2020) and UCSC Genome Browser (Lee *et al.*, 2020) have been developed but are devoted exclusively to eukaryotic genomes. The UCSC Microbial Genome Browser (Chan *et al.*, 2012), EnsemblGenomes (Howe *et al.*, 2020) and MicrobesOnline (Dehal *et al.*, 2010) provide only a limited number of prokaryotic genomes or exclusively bacterial ones and are not updated on a regular basis. The NCBI microbial genome browser (www.ncbi.nlm.nih.gov/genome/microbes/) provides to most comprehensive access to prokaryotic genomes. However, with a searchable organism list over 5000-page-long and a complex horizontal genome viewer, this resource requires a number of user gestures and its results are difficult to export. The retrieval of a particular gene and its immediate synteny from a given prokaryotic genome remains a daunting task for wet bench experimentalists. For this reason, we developed the BAGET web service to provide a free, immediate and effortless access to the sequence, genomic context, intergenic regions and coding capacity of any gene from all complete prokaryotic genomes (Oberto, 2008). Here, we propose the BAGET 2.0 update which exhibits additional features, significant performance improvements and the capacity to handle and provide effortless access to >25 000 prokaryotic genomes.

## 2 Materials and methods

BAGET 2.0 has been completely rewritten in C# 8.0 and .NET Core 3.1 to comply with the industry-standard Model-View-Controller architectural framework and the latest HTML 5.0 specifications. The use of JQuery (jquery.com) and D3 (d3js.org) JavaScript libraries enables asynchronous data transfer and access to the HTML 5.0 graphical canvas, respectively. The BAGET 2.0 database is hosted locally and shared with the FITBAR and SYNTTAX web services (Oberto, 2010, 2013). Incremental database updates occur daily by retrieving new GBFF genomes from the NCBI (ftp.ncbi.nlm.nih.gov) (Sayers *et al.*, 2020). Multiple replicons present in some genomes are accessible separately in BAGET 2.0 and identified by the organism name tag followed by the C1, C2 … Cn suffices, by decreasing size.

## 3 Features

All the features provided by the original BAGET web service have been maintained and several additions and improvements were implemented in BAGET 2.0 as follows:

1. To facilitate user interaction, the initial replicon selection from the database is based on the first letter of the corresponding organism name. A selectable scrolling list of organisms with the same initial is then provided.
2. BAGET 2.0 accepts the submission of user-provided annotated genomes in the GenBank GBFF format. Uploaded genomes are processed in real time and their features are exposed similarly to database entries. User-uploaded genomes are not added to the database and remain confidential.
3. Upon selection or user-uploading of a replicon, a corresponding gene list is created where individual genes can be selected to generate a complete report.
4. Text queries on the annotation of uploaded or database genomes provide a user-selectable gene list.
5. Gene selection generates a proportionally scaled color-coded context map covering a selectable genomic interval between 5 and 30 kb. The gene of interest is depicted in red at the center of the map in 5ʹ to 3ʹ orientation. Surrounding genes in the same orientation are shown in green while the antiparallel genes, in blue. Information on mapped genes is accessible by mouse hovering. Navigation through mapped genes is accessible by mouse clicking.
6. The DNA sequence of the gene of interest and the immediately surrounding genes is shown using the same color-code and genetic orientation as the context map. For protein-coding genes, the colored sequences refer to open reading frames. The intergenic regions are shown in black and gene overlaps are displayed using text background colors. The protein sequence of coding genes in displayed in single letter amino acid code.
7. Each selected coding gene is provided with an external link to the respective protein entry at the NCBI (Sayers *et al.*, 2020) and at Uniprot (UniProt, 2021).
8. PDF reports can be generated either in US-letter or DIN A4 format and downloaded.
9. A contextual help file is accessible through blue question mark icons.

## 4 Results

**Fig. 1.**
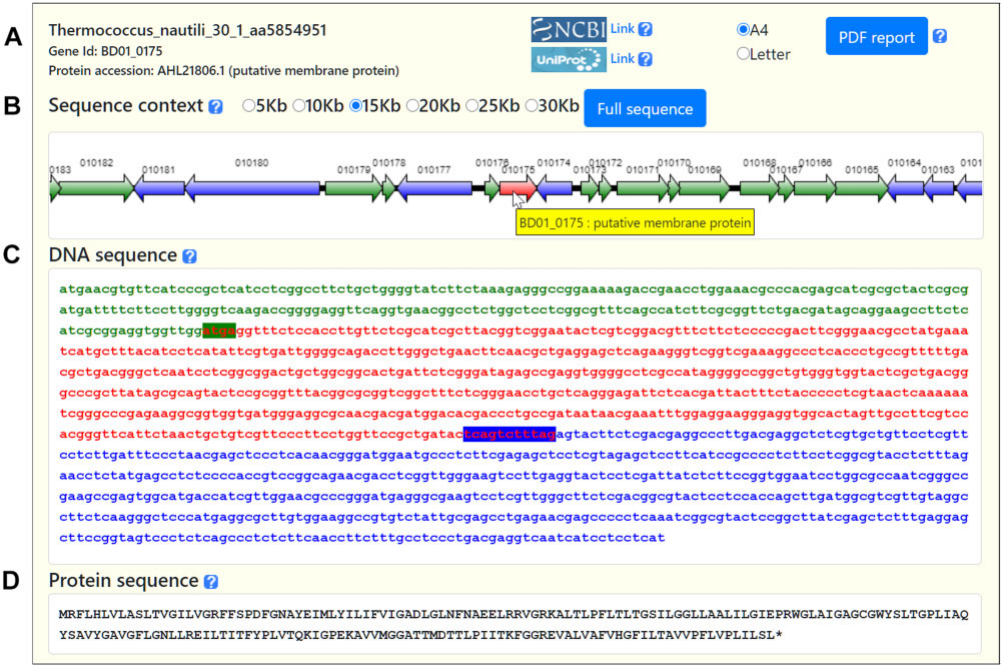
BAGET 2.0 report for the BD01_0175 gene from *T.nautili* 30-1. (**A**) Information relative to the selected gene. (**B**) Graphical context map spanning a user-selectable interval. The gene of interest in shown in the center of the map. (**C**) The DNA sequence of the BD01_0175 open reading frame is shown in red. The extent of gene overlapping with the preceding and following genes are identified by text background color. (**D**) AHL21806.1 protein sequence in amino acid single letter code

BAGET 2.0 provides a fast and effortless access to every gene from over 54 000 replicons corresponding to >25 000 bacterial and archaeal genomes. Its user interface consisting of a single page loaded only once at startup does not require navigation through numbers of forms. In order to mimic user-friendly desktop applications, BAGET 2.0 exchanges data with the server in asynchronous mode with a significant increase in performance. Only three mouse clicks were required to access the BD01_0175 gene sequence and genomic context from *Thermococcus nautili* 30-1 (Fig. 1). The major assets of this web service reside in its user friendliness, daily updated database and quality of the exportable results. To ensure user confidentiality, submitted data, generated results and PDF reports are never saved to disk but reside exclusively in volatile memory for the duration of the user session. BAGET 2.0 does not track users nor use cookies. The versatility and user-friendliness of BAGET 2.0 will benefit wet bench experimentalists and phylogeneticists alike.

## Funding

This work was funded by the ‘Centre Nationale de la Recherche Scientifique, Agence Nationale de la Recherche (grant ANR-19-CE11-0007).

*Conflict of Interest*: none declared.
